# Clinical Evidence Regarding Spermidine–Hyaluronate Gel as a Novel Therapeutic Strategy in Vestibulodynia Management

**DOI:** 10.3390/pharmaceutics16111448

**Published:** 2024-11-12

**Authors:** Filippo Murina, Alessandra Graziottin, Nicla Toni, Maria Teresa Schettino, Luca Bello, Alessandra Marchi, Barbara Del Bravo, Dania Gambini, Lara Tiranini, Rossella Elena Nappi

**Affiliations:** 1 Lower Genital Tract Disease Unit, V. Buzzi Hospital, University of the Study of Milan, 20122 Milan, Italy; 2 Centre of Gynaecology and Medical Sexology, Department of Obstetrics and Gynaecology, San Raffaele Resnati Hospital, 20097 Milan, Italy; a.graziottin@studiograziottin.it; 3 Isola Tiberina Hospital-Gemelli Isola, 00186 Rome, Italy; tonicla@gmail.com; 4 Department of Woman, Child and General and Specialized Surgery, University of Campania “Luigi Vanvitelli”, 80138 Naples, Italy; mariateresa.sche@gmail.com; 5 Ce.Mu.S.S., ASL Città Di Torino, 10128 Turin, Italy; dr.luca.bello@gmail.com; 6 Private Clinic Centro Salute Pelvi (CSP), 10123 Turin, Italy; alessandra.marchi@gmail.com; 7 Studio Del Bravo Private Clinic, 56121 Pisa, Italy; bdelbravo59@gmail.com; 8 Graziottin Foundation for the Management and Treatment of Pain in Women, NPO, 20097 Milan, Italy; gambinica@tiscali.it; 9 Department of Clinical, Surgical, Diagnostic and Pediatric Sciences, University of Pavia, Viale Golgi 19, 27100 Pavia, Italy; lara.tiranini01@universitadipavia.it (L.T.); nappi@rossellanappi.com (R.E.N.); 10 Research Center for Reproductive Medicine and Gynecological Endocrinology—Menopause Unit, IRCCS S Matteo Foundation, 27100 Pavia, Italy

**Keywords:** vulvodynia, vestibulodynia, spermidine, dyaspareunia

## Abstract

**Background:** Vestibulodynia (VBD) represents a summation and overlapping of trigger factors (infections, hormonal disturbances, allergies, genetic aspects, psychological vulnerability, and others) with broad individual variability. As there are no standard treatment options for VBD, the disease is still in need of appropriate therapeutic tools. **Objectives**: A prospective observational trial was performed to confirm the efficacy of a topical gel containing a spermidine–hyaluronate complex (UBIGEL donna™) as either a stand-alone or companion treatment through a multicenter study on a large sample population. **Methods**: For women with VBD (n = 154), the treatment consisted of approximately two months (4 + 4 weeks) of applications according to the posology of UBIGEL. Evaluation of symptoms was performed on relevant clinical endpoints: dyspareunia and vulvovaginal pain/burning by a visual scale (VAS); vestibular trophism by a vestibular trophic health (VeTH) score; vulvoscopy through a cotton swab test; and the level of hypertonic pelvic floor by a physical graded assessment of levator ani hypertonus. **Results**: A total of 154 patients treated with UBIGEL donna™ showed significant improvements across all five evaluated parameters, including pain, dyspareunia, swab test results, muscle hypertonicity, and vestibular trophism. Pain and dyspareunia scores decreased by 46.5% and 33.5%, respectively, while significant improvements were also observed in the other parameters (*p* < 0.0001). These improvements were consistent across various stratifications, including age and disease duration. **Conclusions**: The findings of the present study suggest that UBIGEL donna™ is effective in alleviating pain and dyspareunia, as well as reducing vestibular hypersensitivity in women with VBD. Although UBIGEL donna™ alone cannot serve as a comprehensive substitute for all recommended therapies, we suggest that multimodal therapy strategies may be crucial for attaining substantial improvement in any aspect of the condition.

## 1. Introduction

Vulvodynia is a common type of chronic genital pain in women, with prevalent studies estimating rates between 10% and 28% among reproductive-aged women [1]. Localized provoked vulvodynia at the vestibule, referred to as vestibulodynia (VBD), constitutes the predominant presentation of the condition, accounting for approximately 80% of cases [2,3]. Women with VBD may characterize vulvar pain as burning, stinging, irritation, rawness, and dyspareunia (painful or uncomfortable intercourse). Most patients with VBD characterize their pain as “hot”, “burning”, or “pricking”, noting that the vestibular region is hypersensitive to tactile stimuli (e.g., during sexual intercourse or tampon insertion) and that the pain intensifies with friction. Moreover, the discomfort characteristic of VBD is consistently linked to pelvic floor muscle hyperactivity. This prolonged pattern can result in diminished blood flow to tissues, hyperactive muscles that function improperly, and the development of myofascial trigger points, which may induce localized or radiating pain, along with significant soreness. Neuropathic pain and hypertonicity are multiple and complicated outcomes of aberrant neural development. VBD is probably not a singular disease, but rather a collection of disorders, characterized by vestibular hypersensitivity and pelvic floor hypertonic dysfunction as the common outcome. VBD signifies a confluence and aggregation of several trigger factors (infections, hormone imbalances, allergies, genetic predispositions, and psychological susceptibility, among others), with their significance and dominance differing among individuals [4]. Prior research, encompassing vestibular tissue immunohistochemistry and susceptibility to hormonal contraceptives, has investigated the influence of sex steroids in vestibular pain disorders [5,6]. A study established a correlation between reduced vestibular mucosa thickness in women with VBD and the intensity of burning/pain as well as the severity of dyspareunia [7]. These findings, along with evidence of diminished vaginal levels of steroidogenic metabolites in women with VBD, indicate a trophic disruption of the vestibular mucosa. This may result in modifications to the mucosal architecture and a reduction in the thickness of the vestibular mucosa, rendering the tissue more susceptible to damage and inflammation.

VBD remains largely underserved despite the array of pharmacological (systemic, topical, or local), surgical, psychosocial, and physiotherapy armamentarium applied by vulvar/pelvic pain specialists, either alone or as combined/team-based interventions. 

The research hypothesis for the present study was to prospectively confirm the efficacy and safety of a topical gel containing a spermidine–hyaluronate complex (UBIGEL donna™) in patients with VBD. The gel’s biological rationale is based on biostimulation through mechanotransduction elements coupled with antioxidant activity on genitalia and with autophagy, which cooperate to support early clinical outcomes of restored integrity of compromised and hyper-reactive vulvovaginal tissues.

Genitourinary tissues contain spermidine, a ubiquitous regulatory molecule with a pivotal role in many cellular events [8]. Notably, “spermidine” was named after its discovery in human semen, along with spermine [9], and was later found conditionally essential in the reproductive physiology of both genders [10]. 

The centrality of spermidine in the complex polyamine biochemical pathway [6] plays a key role in cellular homeostasis, as its scarcity determines tissue sufferance, growth retardation, and involution to senescent phenotype [11]. In physiological conditions, spermidine is prevalently associated with anionic macromolecules, such as DNA and RNA, wherein it forms supramolecular complexes (SMCs) [12]. 

A synthetic SMC of spermidine with polyanionic polymers triggers potent reparative processes on connective tissues over a broad (nM to mM) concentration range. These regenerative effects possibly rely on the sustained release from SMCs of nanomolar amounts of spermidine, otherwise rapidly metabolized or negatively affecting local trophism if released at a higher concentration, as spermidine is highly bioavailable [13]. 

In prior research, it was discovered that spermidine–hyaluronate’s trophic regenerative effects on female genitalia tissues occur via the creation of a trophic microenvironment with local biostimulation of the female genitalia, i.e., as an enhanced version of pure hyaluronan, also assisted by antioxidant protection from oxidative stress [14,15,16]. 

An early pilot study on UBIGEL donna™ revealed a significant reduction in pain and dyspareunia in patients with VBD [17]. The aim of this trial was to confirm the efficacy of UBIGEL donna^™^ in VBD by a multicenter study on a larger sample population. 

## 2. Materials and Methods

### 2.1. Trial Design

This was a prospective, observational trial. Each eligible subject participated in this study for approximately 2 months. This study was approved by the ethics committee of the coordinator center, and all women provided informed written consent (V. Buzzi hospital EC-n. 2236/24-06/0272024).

### 2.2. Study Population

The patient population included women with VBD.

#### 2.2.1. Subject Inclusion Criteria

All criteria below were met by subjects eligible for study participation.

- Women at least 18 years of age who had not experienced menopause (an absence of menstruation for 12 months);
- Experienced moderate to severe pain (minimum 5/10 on a numerical rating scale) in at least 90% of attempted sexual intercourse;
- Pain limited to the vestibule during vaginal intercourse and/or during activities exerting pressure on the vestibule (tampon insertion, tight jeans or pants, cycling, and horseback riding);
- Presence of VBD for at least 3 months, diagnosed according to the standardized gynecological examination protocol by a staff gynecologist;
- Willing to attempt sexual activity between visits.

#### 2.2.2. Subject Exclusion Criteria

Subjects who met any of the following criteria were excluded.

- Active vulvovaginal infections at the time of their gynecological examination;
- Genital bleeding of unknown origin;
- Patients concomitantly included in different interventional clinical trials;
- Women who were using or had used topical drugs in the past 30 days;
- Women with concomitant vulvar dermatosis or other vulvar disorders.

### 2.3. Clinical Outcomes

The primary efficacy outcome included evaluation and changes assessed as follows:

- A 0–10-point visual scale (VAS) related to vulvar burning/pain and dyspareunia;
- A vestibular cotton swab test (a small cotton-tipped applicator lightly rolled over surfaces of the vestibule (mean values at 1, 3, 5, 6, 7, 9, and 11 o’clock locations by asking the subject to report pain intensity on a discrete visual analog scale of 1 (no pain) to 10 (worst possible pain);
- Vestibular trophism evaluated through a validated specific vestibular trophic health (VeTH) score [18]. The VeTH evaluation considered five elements related to vestibular trophism: petechiae, pallor, thinning, dryness, and redness. The intensity of each characteristic was rated on a scale from 0 to 3 (0 = none; 1 = mild; 2 = moderate; and 3 = severe) and summed to provide a total VeTH score ranging from 0 to 15, with lower scores indicating better VeTH;
- Clinical evaluation of hypertonic pelvic floors required physical exams documenting the hypertonus of the levator ani complex by an experienced examiner. We used an empirical score that allowed reproduction of the pelvic floor hypertonus with acceptable reliability (grade 0 = no hypertonicity; grade 1 = mild hypertonicity; grade 2 = moderate hypertonicity; and grade 3 = severe hypertonicity).

### 2.4. Treatment

Subjects meeting inclusion criteria received a dispenser system containing UBIGEL donna™ gel, and were trained to apply a fingertip unit of the gel to the vulvar vestibule (a fingertip unit = the amount needed to squeeze a line from the tip of an adult finger to the first crease of the finger) three times weekly for 4 weeks, then twice a week for the next 4 weeks, before bedtime. The efficacy of outcomes was evaluated after 60 days of treatment.

### 2.5. Statistical Methods

The distribution of data was initially assessed using the Shapiro–Wilk test, which indicated that data were not normally distributed. Consequently, non-parametric statistical methods were employed for analysis. To evaluate pre-treatment (V1) and post-treatment (V2) differences within the group of patients, the Wilcoxon signed-rank test for paired data was used. The Kruskal–Wallis test was then employed to determine differences in treatment responses when stratified by various parameters. Additionally, a two-way ANOVA was also used to confirm the effect on stratifications, as detailed in the Supplementary Materials. All analyses were performed using GraphPad 8.0.2 software, and the level of significance for all tests was set at 0.05.

#### Determination of Sample Size

We used the http://statulator.com program (accessed on 4 January 2024), which calculated the sample size for paired differences with a power of 80% and a level of significance of 5%, to detect the mean of the differences of the VAS scale value of 1.5 (20%) between pairs. Assuming the standard deviation of the differences to be 2, we needed to recruit almost 80 participants.

## 3. Results

A total of 154 patients participated in and completed this study. The only reported side effect was a mild stinging discomfort at application in 12 of the 154 women (7%), with discontinuation for only 2–3 days. 

Specifically, the average age of patients was 31 years, with an illness duration averaging 55 months. Recurrence of UTIs was observed in 62% of patients, while 36% experienced recurrent vaginitis. Regularity of menstrual cycles was reported in 70% of participants. Among these patients, 54% had previous pregnancies, 38% had undergone prior treatments, and 84% were receiving ongoing treatment at the time of this study. Previous use of contraception was reported by 49% of participants, with ongoing contraceptive use noted in 38%. The average duration of contraceptive use among participants was 28 months.

**Figure 1:**
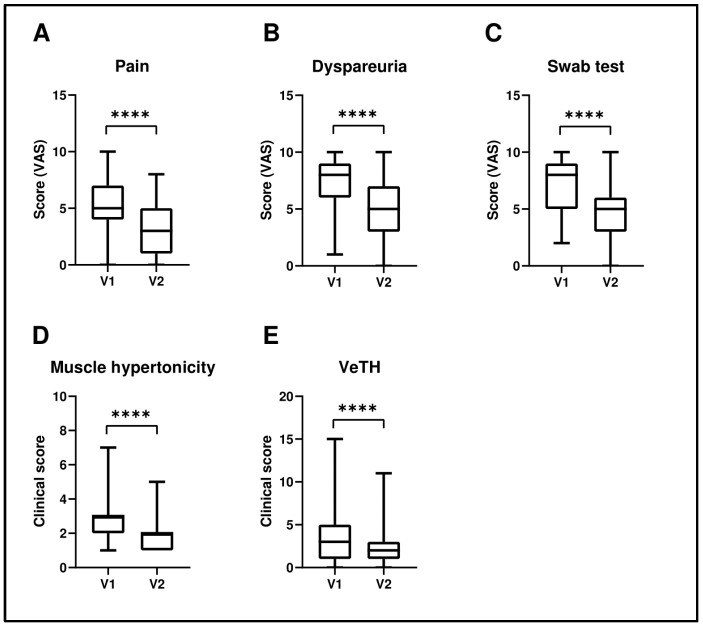
Comparison of pre-treatment (V1) and post-treatment (V2) data for pain (**A**), dyspareunia (**B**), swab test (**C**), muscular hypertonicity (**D**), and vestibular trophism (**E**). Statistical analysis was performed using the Wilcoxon signed-rank test for paired data. **** *p* < 0.0001.

Specifically, as reported in Table 1 and Figure 1, pain decreased from a mean score of 5.08 ± 3.00 (V1) to 3.10 ± 2.34 (V2), representing a 46.5% improvement (*p* < 1 × 10^−10^). Dyspareunia decreased from 7.45 ± 2.06 to 4.95 ± 2.52, reflecting a 33.5% improvement (*p* < 1 × 10^−18^). Swab test results improved from 7.10 ± 2.02 to 4.81 ± 2.12, showing a 32.2% improvement (*p* < 1 × 10^−19^). Muscle hypertonicity was reduced from 2.62 ± 0.98 to 1.77 ± 0.83, indicating a 32.5% reduction (*p* < 1 × 10^−15^). Finally, vestibular trophism improved from 3.40 ± 2.85 to 2.26 ± 1.96, corresponding to a 33.5% improvement (*p* < 0.000067).

**Figure 2:**
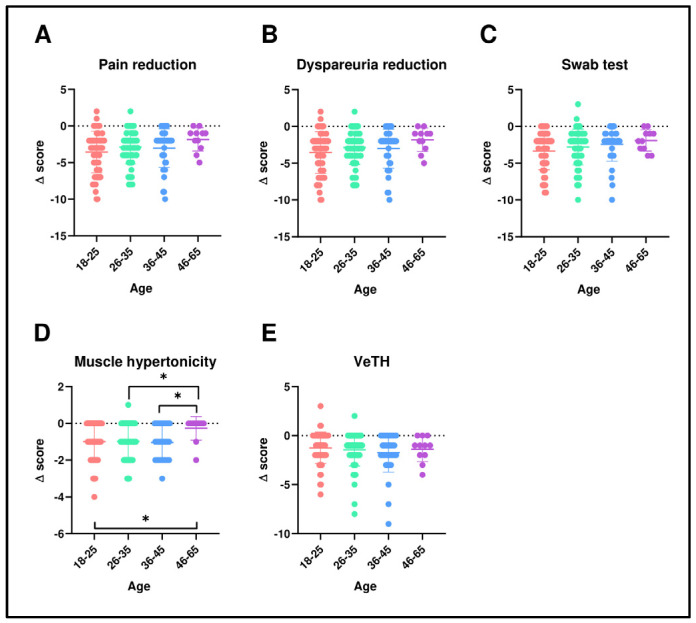
Comparison of deltas stratified by age groups for pain (**A**), dyspareunia (**B**), swab test (**C**), muscular hypertonicity (**D**), and vestibular trophism (**E**). Statistical analysis was performed using the Wilcoxon signed-rank test for paired data. * *p* = 0.05.

**Figure 3:**
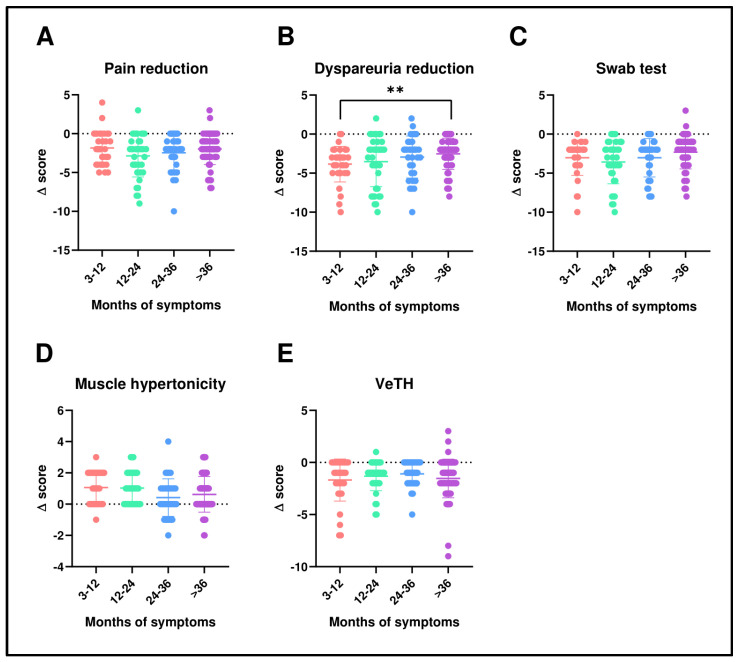
Comparison of deltas stratified by months of symptoms groups for pain (**A**), dyspareunia (**B**), swab test (**C**), muscular hypertonicity (**D**), and vestibular trophism (**E**). Statistical analysis was performed using the Wilcoxon signed-rank test for paired data. ** *p* < 0.01.

Figure 2A–E and Figure 3A–E illustrate the distribution of delta values (d = V2 − V1) representing the treatment’s efficacy. These figures show that improvements were consistent across various strata, including age and disease duration. Further analyses of absolute values, reported in Supplementary Figures S1–S5, based on different strata such as age, disease duration, previous treatments, current contraception, and recurrent infections or vaginitis, were conducted. 

As shown in Figure S3E, stratification did not maintain statistical significance only for vestibular trophism in cases with more than nine therapies, while other parameters continued to exhibit significant improvements.

## 4. Discussion

These results demonstrate significant improvements across all five evaluated parameters (pain, dyspareunia, swab test, muscle hypertonicity, and vestibular trophism) after the use of UBIGEL donna™. Moreover, improvements were consistent through multiple layers, including age and disease duration. 

The pattern of VBD responses indicates sensory anomalies characterized by evoked pain (e.g., hyperalgesia or allodynia), implying sensitization, a fundamental feature of neuropathic pain. This aligns with biopsy studies indicating heightened innervation of the vulvar vestibule, along with elevated subepithelial heparinase activity and cytokines associated with neuroinflammatory processes; patients with VBD also report alterations in sensitivity, implying that sensory dysregulation may contribute to the manifestation of this pain condition [3].

Spermidine is a naturally produced polyamine of low molecular weight, prevalent among several organisms. It has three positively charged amino groups, and this structural feature is essential for its actions, which encompass stabilizing DNA, facilitating cell development, and initiating autophagy [19]. A high spermidine physiologic content positively correlates with healthy status, including lifetime extension, tumor suppression, cardiovascular protection, and neuromodulation.

What factors contribute to spermidine’s effectiveness in VBD?

Several pathophysiological factors, such as an increase in nerve fiber density in the vulvar vestibule and a localized inflammatory response, are deemed crucial in the onset and persistence of VBD [20]. Tissue from the afflicted regions exhibited invading immune cells and inflammatory mediators (e.g., mast cells and cytokines) indicative of an inflammatory response. A definitive connection exists between pain and inflammation; inflammation and proinflammatory mediators have long been correlated with allodynia, the primary characteristic of neuropathic pain. In VBD, a recurrent trigger factor, such as a vulvovaginal infection, elicits an excessive inflammatory response, which activates pain signals by augmenting the quantity of vestibular nerve fibers. 

Researchers are progressively investigating the influence of hormonal imbalances on the onset of VBD. An ultrasound assessment of the vestibular mucosa indicated reduced thickness relative to healthy women of equivalent age, exhibiting a value nearly comparable to that observed in postmenopausal women [7], hence rendering the vestibular mucosa more susceptible to nociception. Moreover, certain investigations indicated that the expression of estrogen and androgen receptors in VBD patients may be both qualitatively and quantitatively compromised [21,22].

We may correlate the positive results from our study on the topical use of spermidine in VBD to its trophic action, anti-neuropathic effects, and modulation of inflammation.

A recent study found that topical spermidine therapy greatly enhanced skin wound healing by activating a specific route. Furthermore, local spermidine prompted an expedited modulation in cytokine expression following a skin wound, suggesting that spermidine may play a role in enhancing the trophic status associated with wound healing [23]. Spermidine’s trophic function influences tubulin assembly, an essential element of cytoskeletal proteins and cellular structure. Microtubules (MTs) are cylindrical polymers of cytoskeleton formed from tubulin heterodimers, whose dynamic characteristics are crucial for various cellular functions, including cytoplasmic organization, polarity establishment, morphogenesis, and the maintenance of appropriate trophism [24]. Certain investigations have shown that spermidine possesses significant capabilities by moderating the release of chemokines and cytokines, leukocyte infiltration, and epithelial damage [16]. Moreover, the diverse attributes of spermidine include its antioxidant and free radical scavenging capabilities in support of its neuroprotective action [25]. Spermidine alleviated neuropathic pain by reducing oxidative stress and enhancing histological alterations and behavioral assessments in a live model of injury-induced, chronic neuropathic pain [26].

The use of a specific formulation of spermidine SMC formed between spermidine and polyanionic polymers like hyaluronic acid (HA), alginate, and the like, at variable spermidine concentrations and a fixed polymer level, may further support our findings. Spermidine SMC speeds up cell growth across the whole concentration range, with up to 80% of its power being available at sub-μmol/L levels. Spermidine HA also increased the expression of Ki-67 in reconstituted human vaginal epithelium [13], providing proof of local regenerative effects, while safety data for this organoid model justifies the use of spermidine SMC in atrophic disorders. It was also demonstrated that not all spermidine concentrations were likewise effective in reducing the release of noxious stimuli from macrophages, hence suggesting a dose-dependent mode of action of natural polyamines [27]. Notably, results obtained in this multicenter study almost overlap with those from a preliminary study by UBIGEL donna™ on VBD [4], providing reproducibility proof of the product’s efficacy. 

Although lack of a follow-up without treatment bias and the absence of placebo effect may account for some of the observed benefit, we doubt that these could account for the substantial benefit seen in most patients. Another potential weakness of our study included the potential function of HA combined with spermidine; HA’s moisturizing and soothing properties may have contributed to the positive results achieved.

This series leads us to believe that a randomized controlled trial is necessary to validate these promising outcomes. In comparison to other treatments used for VBD to date, this study shows that topical spermidine–hyaluronate has the potential to assist both pain and the inability to conceive at the same time, while still being well tolerated and accepted by patients.

In conclusion, our study demonstrated that UBIGEL donna™ provides significant therapeutic benefits for patients, notably in reducing pain and dyspareunia. Additionally, improvements across other clinical parameters—including swab test results, muscle hypertonicity, and vestibular trophism—indicated a comprehensive positive effect of this treatment. These outcomes suggest that UBIGEL donna™ could be a valuable intervention for enhancing patient quality of life and addressing diverse symptoms associated with VBD, offering a promising approach in the management of similar cases in clinical practice.

## Figures and Tables

**Table 1:** Treatment efficacy of UBIGEL donna™.

	Pain	Dyspareunia	Swab Test	Muscle Hypertonicity	VeTH
Basal (V1)	5.08	7.45	7.10	2.62	3.40
Follow up (V2)	3.10	4.95	4.81	1.77	2.26
SD (V1)	3.00	2.06	2.02	0.98	2.85
SD (V2)	2.34	2.52	2.12	0.83	1.96
Improvement (%)	46.5	33.5	32.2	32.5	33.5
*p*-value	<0.0001	<0.0001	<0.0001	<0.0001	<0.0001

## Data Availability

The original contributions presented in the study are included in the article/Supplementary Materials, further inquiries can be directed to the corresponding authors.

## Supplementary Materials

The following supporting information can be downloaded at: https://www.mdpi.com/article/10.3390/pharmaceutics16111448/s1, Figure S1: Comparison of pre-treatment (V1) and post-treatment (V2) stratified data by age for pain, dyspareunia, swab test, muscular hypertonicity, and vestibular trophism. Figure S2. Comparison of pre-treatment and post-treatment stratified data by months of symptoms for pain, dyspareunia, swab test, muscular hypertonicity, and vestibular trophism. Figure S3. Comparison of pre-treatment (V1) and post-treatment (V2) stratified data by months of contraception for pain, dyspareunia, swab test, muscular hypertonicity, and vestibular trophism. Figure S4. Comparison of pre-treatment (V1) and post-treatment (V2) stratified data by urinary tract infection for pain, dyspareunia, swab test, muscular hypertonicity, and vestibular trophism. Figure S5. Comparison of pre-treatment (V1) and post-treatment (V2) stratified data by recurrent vaginitisfor pain, dyspareunia, swab test, muscular hypertonicity, and vestibular trophism.
