# Supplementary material for: Clinical Evidence Regarding Spermidine–Hyaluronate Gel as a Novel Therapeutic Strategy in Vestibulodynia Management
Source: Pharmaceutics. 2024 Nov 12;16(11):1448. doi: 10.3390/pharmaceutics16111448 (PMC11597842; doi:10.3390/pharmaceutics16111448)
Supplement: Supplementary file 1 [file pharmaceutics-16-01448-s001.zip › pharmaceutics-3269253-supplementary.pdf]

Supplementary material

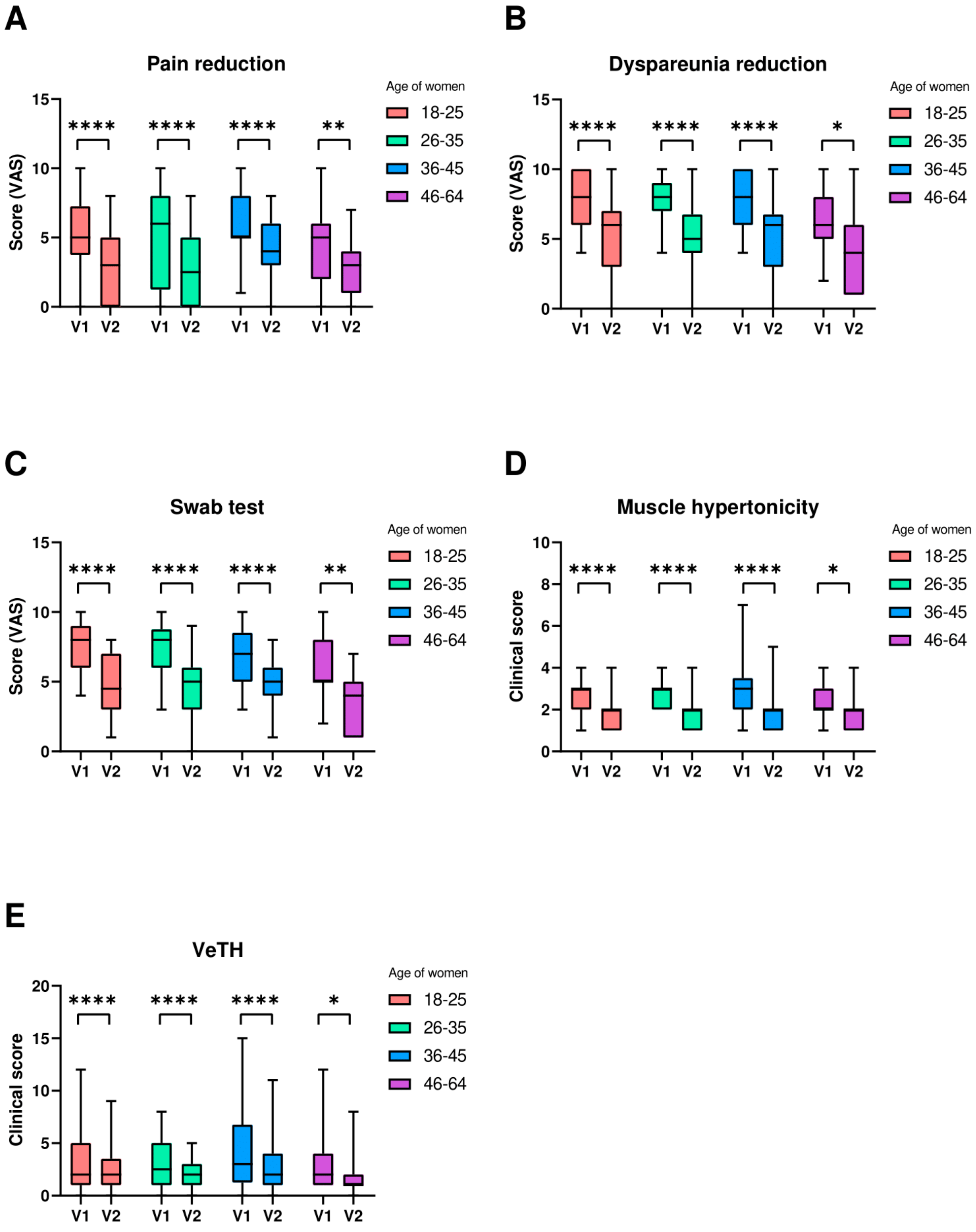

Figure S1. Comparison of pre-treatment (V1) and post-treatment (V2) stratified data by age for pain (A), dyspareunia (B), swab test (C), muscular hypertonicity (D), and vestibular trophism (E). Statistical analysis was performed using the Wilcoxon signed-rank test for paired data. **** $p < 0.0001$; ** $p<0,01$; *$p=0,05$.

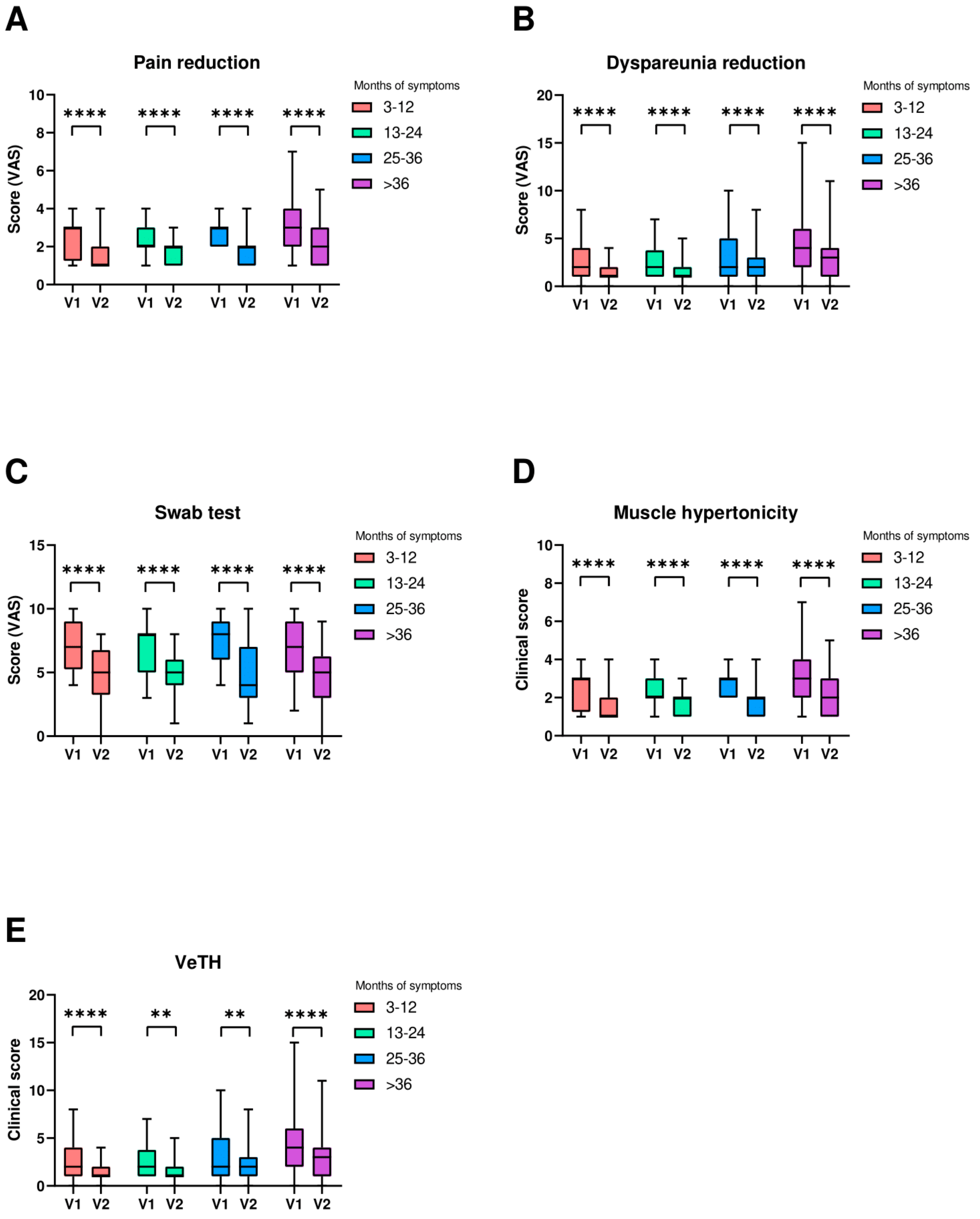

Figure S2. Comparison of pre-treatment (V1) and post-treatment (V2) stratified data by months of symptoms for pain (A), dyspareunia (B), swab test (C), muscular hypertonicity (D), and vestibular trophism (E). Statistical analysis was performed using the Wilcoxon signed-rank test for paired data. **** $p < 0.0001$; ** $p<0{,}01$.

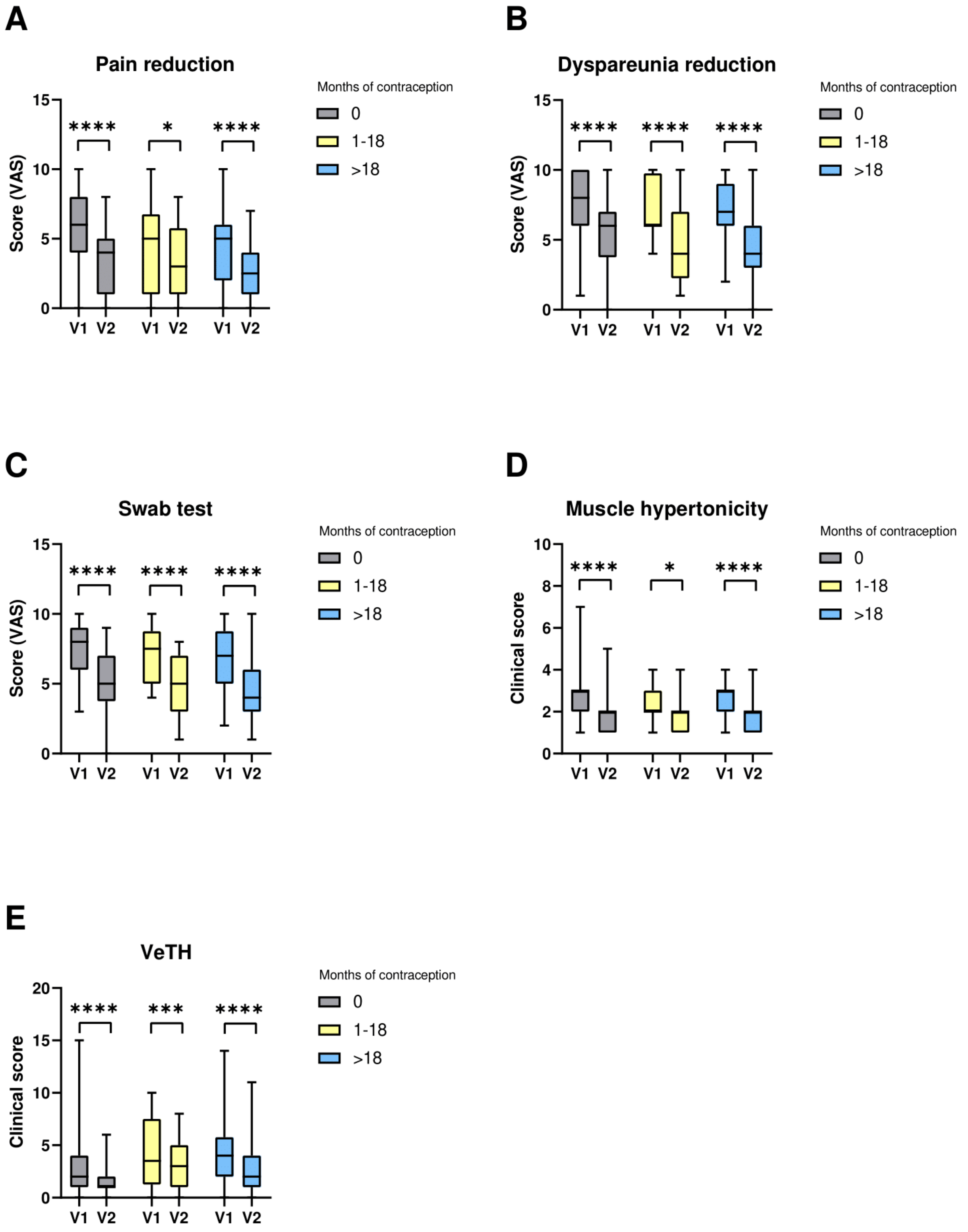

Figure S3. Comparison of pre-treatment (V1) and post-treatment (V2) stratified data by months of contraception for pain (A), dyspareunia (B), swab test (C), muscular hypertonicity (D), and vestibular trophism (E). Statistical analysis was performed using the Wilcoxon signed-rank test for paired data. **** $p < 0.0001$.

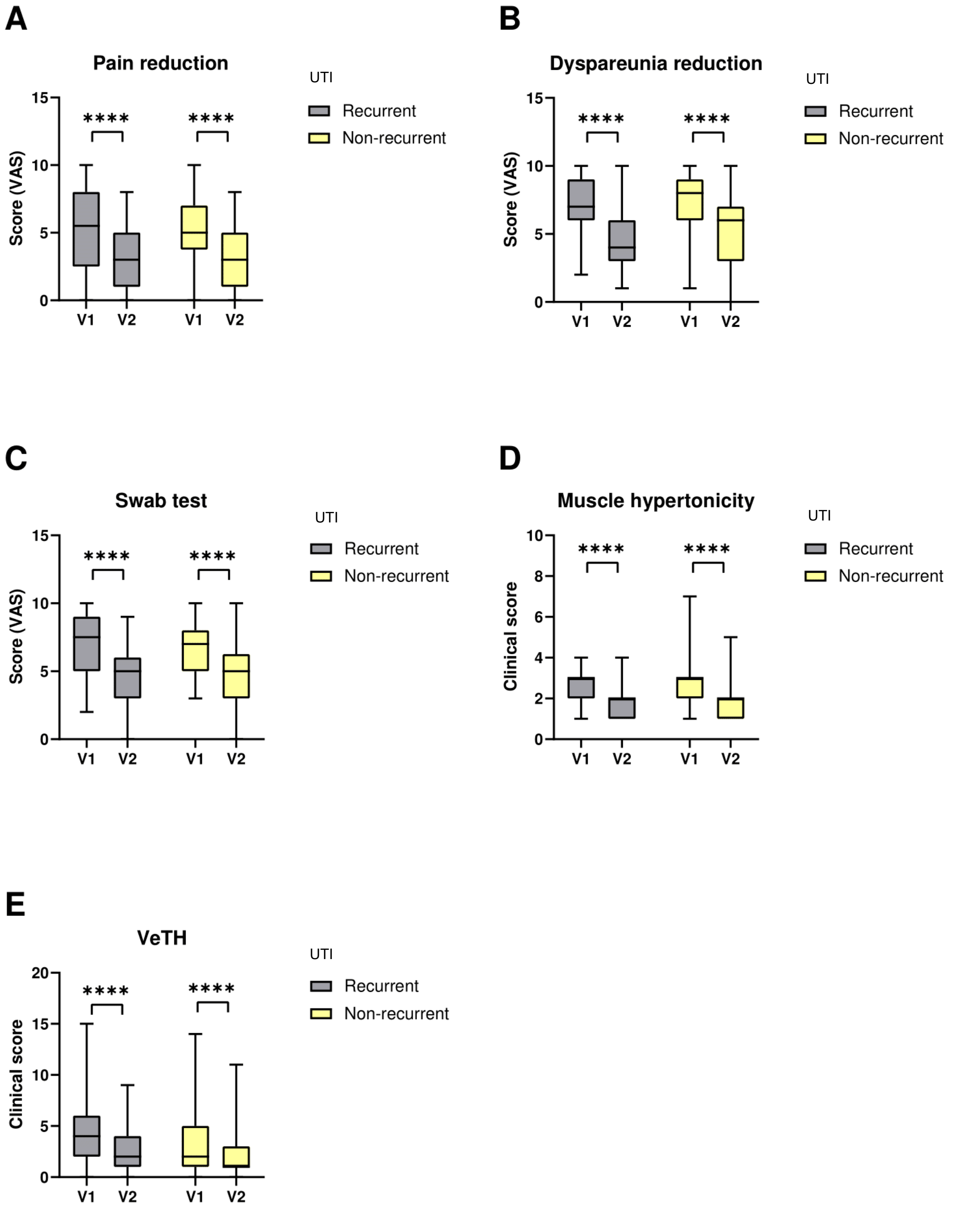

Figure S4. Comparison of pre-treatment (V1) and post-treatment (V2) stratified data by Urinary Tract Infection for pain (A), dyspareunia (B), swab test (C), muscular hypertonicity (D), and vestibular trophism (E). Statistical analysis was performed using the Wilcoxon signed-rank test for paired data. **** $p < 0.0001$.

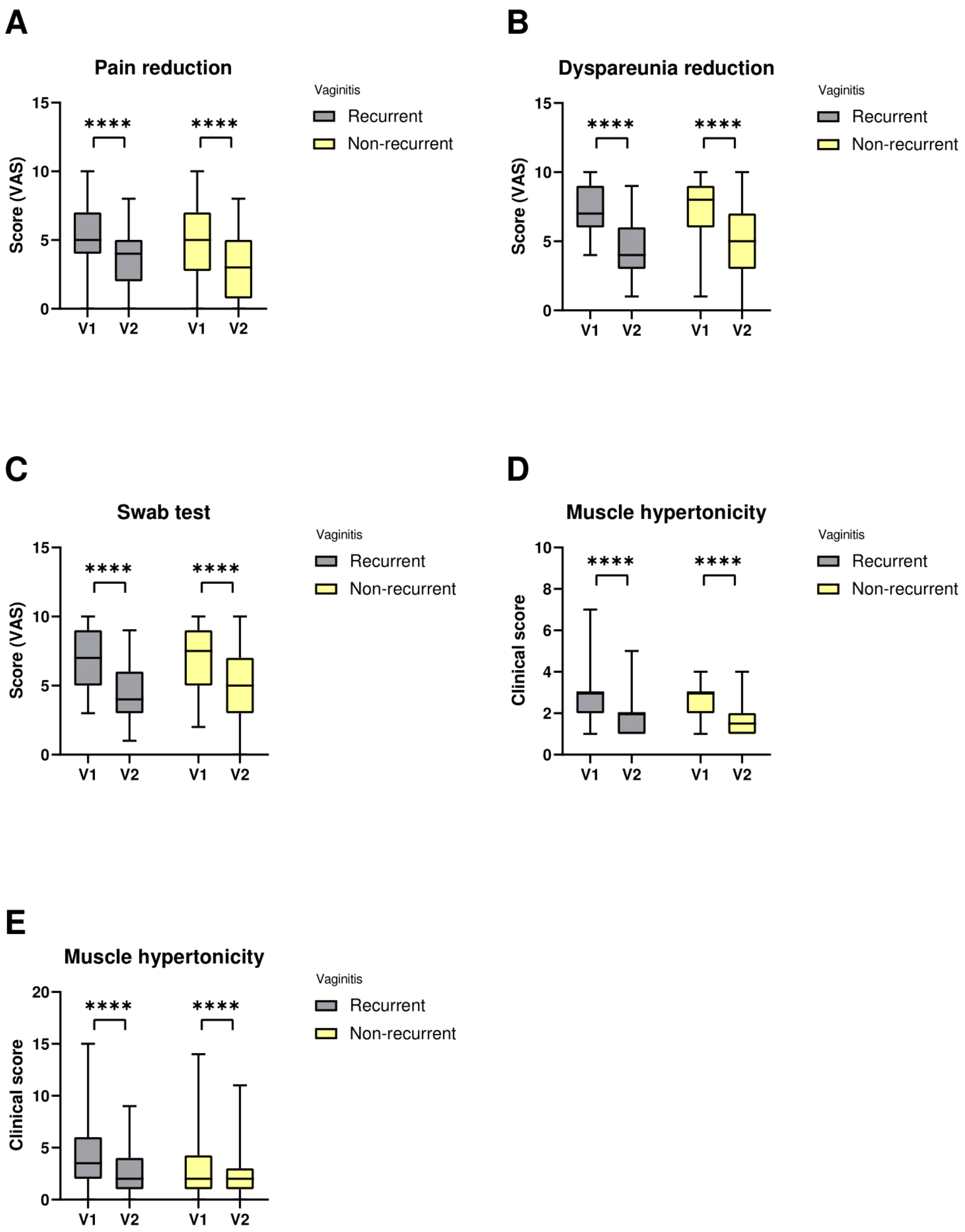

Figure S5. Comparison of pre-treatment (V1) and post-treatment (V2) stratified data by recurrent vaginitis for pain (A), dyspareunia (B), swab test (C), muscular hypertonicity (D), and vestibular trophism (E). Statistical analysis was performed using the Wilcoxon signed-rank test for paired data. **** $p < 0.0001$.
